# Demonstration of a SiC Protective Coating for Titanium Implants

**DOI:** 10.3390/ma13153321

**Published:** 2020-07-26

**Authors:** Chaker Fares, Shu-Min Hsu, Minghan Xian, Xinyi Xia, Fan Ren, John J. Mecholsky, Luiz Gonzaga, Josephine Esquivel-Upshaw

**Affiliations:** 1 Chemical Engineering Department, College of Engineering, University of Florida, Gainesville, FL 32611, USA; c.fares@ufl.edu (C.F.); mxian@ufl.edu (M.X.); fren@che.ufl.edu (F.R.); 2 Department of Restorative Dental Sciences, Division of Prosthodontics, College of Dentistry, University of Florida, Gainesville, FL 32610, USA; shuminhsu@ufl.edu (S.-M.H.); xiaxinyi@ufl.edu (X.X.); 3 Materials Science and Engineering, College of Engineering, University of Florida, Gainesville, FL 32611, USA; jmech@mse.ufl.edu; 4 Center for Implant Dentistry, College of Dentistry, University of Florida, Gainesville, FL 32610, USA; lgonzaga@dental.ufl.edu

**Keywords:** biomaterials, coating, implant, dentistry

## Abstract

To mitigate the corrosion of titanium implants and improve implant longevity, we investigated the capability to coat titanium implants with SiC and determined if the coating could remain intact after simulated implant placement. Titanium disks and titanium implants were coated with SiC using plasma-enhanced chemical vapor deposition (PECVD) and were examined for interface quality, chemical composition, and coating robustness. SiC-coated titanium implants were torqued into a Poly(methyl methacrylate) (PMMA) block to simulate clinical implant placement followed by energy dispersive spectroscopy to determine if the coating remained intact. After torquing, the atomic concentration of the detectable elements (silicon, carbon, oxygen, titanium, and aluminum) remained relatively unchanged, with the variation staying within the detection limits of the Energy Dispersive Spectroscopy (EDS) tool. In conclusion, plasma-enhanced chemical vapor deposited SiC was shown to conformably coat titanium implant surfaces and remain intact after torquing the coated implants into a material with a similar hardness to human bone mass.

## 1. Introduction

The use of titanium in dental and orthopedic implants has been well reported in the literature. Since the first studies reported by Brånemark, significant enhancements have been made to the structure, chemistry, and procedure of placing titanium implants [1,2,3,4,5]. In recent years, various surface treatments and designs of titanium implants have been proposed by research groups and companies to improve the survivability of these implants [4]. In addition to these enhancements, alternative materials such as zirconia have been introduced in implant dentistry [6,7,8,9]. Despite these alternative materials, titanium remains the most popular material for implants due to this material’s versatility in design, morphology, and connection [4,10,11].

In the dental field, commercially pure titanium (cp Ti) and titanium alloys are utilized as implants due to their relatively high corrosion resistance, osseointegration, strength, and biocompatibility [12,13,14,15]. One study showed that the clinical success rate for titanium-based implants is 87.8% over a 36-year follow up period [10]. The foundation of this success rate is rooted in the long-term stability of the implant–bone surface and the osseointegration capability of the implant [1,3,10,16,17,18]. Although the survivability of these implants has increased, there is still room for improvement to minimize the corrosion of titanium.

This osseointegration capability and the corrosion resistance of titanium-based implants has been attributed to the formation of a protective oxide film that forms on the implant when in contact with the surrounding environment [13,19,20,21,22]. Despite the protective nature of the native oxide film, there are factors within the oral environment that can degrade this oxide layer and cause a release of ions that can be toxic to oral tissues [23,24,25,26,27,28]. Some of the corrosive substances that can cause metallic degradation are citric acid, lactic acid, hydrogen peroxide, hydrochloric acid, and hydrofluoric acid at different concentrations. Within the saliva, ions such as H^+^, F^−^, and Cl^−^ are the main elements responsible for dental material corrosion [25,26]. The degree of corrosion depends on many factors such as a patient’s oral hygiene, a patient’s diet, dental treatments, oral biofilm composition, as well as the composition and flow of a patient’s saliva [13,27,29,30]. 

Within the literature, there are reports highlighting the stability of titanium and Ti alloys, while other reports mention various complications associated with titanium implants over time. These studies can be conflicting to the reader and therefore both sides will be mentioned in this report. In 2018, the Consensus Conference of the European Association for Osseointegration (EAO) stated in its final report that (1) the evidence for titanium hypersensitivity is weak, (2) the clinical impact of titanium particles on tissues is “unclear”, and (3) there is no evidence to prove the causative role of Ti particles in periimplantitis [31]. On the other hand, one group found that the degradation of titanium and subsequent release of metallic ions into tissues can cause an inflammatory response and even mutagenic or carcinogenic reactions in severe cases [32,33,34,35,36,37,38,39]. Additionally, a significant correlation has been reported between alveolar bone loss around titanium implants and peri-implant inflammation [33,34]. Regardless of the conflicting nature of these reports, additional research is needed to understand potential risks and improvement points more clearly for titanium-based materials. Additionally, it is evident that minimizing the corrosion of titanium implants should be a critical focus for future research. However, the corrosive resistance of titanium implants must not be developed at the cost of osseointegration. Both corrosion resistance and osseointegration should be considered when modifying the titanium surface, as these parameters are critical in ensuring implant success and survival within a patient’s oral cavity. 

To improve osseointegration rates, modification of implant surfaces such as acid-etching, grit-blasting, and organic–inorganic surface treatments have been commercially adopted [17,18,40,41,42]. Other viable methods have included altering the titanium surface at the nanoscale level to stimulate the osteogenic cell migration process [43,44]. In a recent study, Maminskas et al. demonstrated that novel bioceramic ceramic coatings may be promising for biomedical applications due to their measured biocompatibility and human gingival fibroblast adhesion [45]. Although these innovations have enhanced the osseointegration capability of titanium implants, they have not directly addressed corrosion mitigation. Another possible alternative to direct surface modification is coating the titanium implant with a protective material to reduce corrosion effects and their associated side effects. Out of many potential materials that could protect titanium from corrosion, silicon carbide (SiC) seems promising due to this material’s high strength, lack of reactivity to the oral environment, and ease of deposition [46,47,48,49,50,51,52,53,54,55]. Using SiC as a protective coating has proven to be effective for glass-ceramics used for fixed dental prostheses and significantly reduced the effects of corrosion [54,56]. In addition to SiC’s durability, this coating has also shown favorable biocompatibility in various applications [57,58,59,60,61,62,63]. Furthermore, a report by Camargo et al. illustrated that a protective SiC coating used for dental ceramics could be used to mitigate the detrimental effects of ceramic corrosion over time [64]. Based on these previous reports, one of the aims of this study is to determine if the demonstrated SiC coating on dental ceramics can also be utilized for titanium-based materials. Although these initial reports have shown the promise of SiC-based titanium coatings, significant work is still needed to optimize the adhesion of the SiC film and ensure that the coating osseointegrates in the patient’s oral cavity. The SiC coating must remain intact during and after torquing the implant into bone to maximize this material’s beneficial properties.

In this study, we demonstrate the ability of PECVD SiC to remain intact on titanium implants after simulated implant placement using scanning electron microscopy (SEM), energy dispersive spectroscopy (EDS), Fourier transform infrared spectroscopy (FTIR), and X-ray photoelectron spectroscopy (XPS).

## 2. Materials and Methods 

### 2.1. Experimental Design

Prior to depositing SiC onto titanium implants, a pilot study was performed by depositing SiC onto high-purity titanium disks (2 × 11 mm^2^). To accomplish this, the disks were coated with SiC, ground using a chewing simulator to expose the SiC/Ti interface, and then examined using SEM to determine the adhesion and interfacial quality. 

To determine if a SiC protective coating on titanium implants can withstand clinical implant placement, the following steps were performed. First, titanium implants (Imtec, 1.8 × 10 mm^2^) were examined under SEM to characterize their morphology prior to any SiC coating. Next, the same implants were coated with SiC and examined again using SEM, EDS, and XPS. Lastly, these SiC-coated implants were torqued into an acrylic block to simulate clinical implant placement. After the implants were removed from the block, the surfaces were examined a third time to determine if the SiC coating remained intact. 

**Figure 1:**
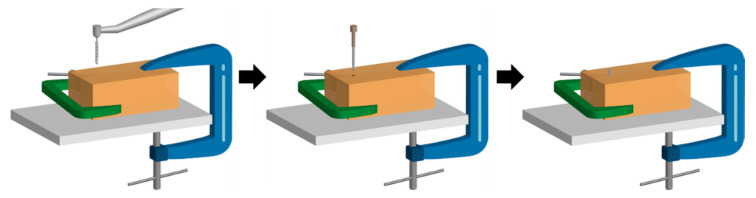
Schematic process of how the SiC-coated titanium implants were torqued into acrylic blocks to simulate implant placement.

After coating the implants with SiC, the implants were torqued into polymethylmethacrylate (PMMA) blocks to determine if the SiC coating could withstand the placement process. PMMA was selected due to its similar hardness to human bone mass. Figure 1 shows a graphical schematic of how the SiC was placed into the PMMA block. First, two blocks of PMMA were clamped together so that the implant could be removed after torquing while preserving the surface. Next, a small diameter pilot drill was used on a slow-speed handpick to start the implant placement. Then, the SiC-coated implants were manually torqued at the manufacturer recommended value of 35 N. After implant placement, the PMMA blocks were separated to gently remove the coated implant for characterization. PMMA blocks (22 × 8 × 15 mm^3^) were utilized to simulate implant placement into a hard surface similar to bone to quantify if the proposed SiC coating could withstand the torquing process. The PMMA blocks were fabricated using ortho acrylic resin powder (Orthodontic Resin Clear, Henry Schein, Melville, NY, USA) and caulk orthodontic resin (Dentsply, Dentsply Sirona, York, PA, USA). Pig jaw bones are traditionally used for clinician implant training; however, two blocks of PMMA were selected so that the blocks could be separated after placement to remove the implant and examine the interface. PMMA has a Shore D hardness of 96, which is comparable to the hardness of bone at 101 on the same scale [65,66,67,68,69].

### 2.2. SiC Coating

To coat the titanium disks and implants used in this study, a plasma-enhanced chemical vapor deposition system (PECVD, PlasmaTherm 790, Saint Petersburg, Russia) consisting of a parallel plate configuration, gas shower head, and load lock was used to deposit 20 nm of silicon dioxide (SiO_2_) and 200 nm silicon carbide (SiC) onto the samples. The SiO_2_ layer between the titanium and SiC was used to improve adhesion. Nitrous oxide (N_2_O) and silane (SiH_4_) were used as gas precursors for SiO_2_ deposition. For the silicon carbide deposition, silane and methane (CH_4_) were the gas precursors. The deposition temperature was 300 °C and the rates of deposition were 330 Å/min and 170 Å/min for the SiO_2_ and SiC, respectively. The radio frequency (RF) power for the SiO_2_ and SiC deposition was 30 W and 400 W, respectively, at a frequency of 13.56 MHz. The chamber pressure for SiO_2_ and SiC deposition was 800 and 1100 mTorr, respectively. The self-bias voltage was between 0 and 3 V for the SiO_2_ film and between 15 and 20 V for the deposited SiC. The PECVD rate was regularly verified by depositing SiO_2_ and SiC onto reference wafers and measuring the total thickness using optical and physical profilometry techniques. For the total 220 nm coating utilized in this study, the variation from batch to batch was measured to be within 1.3% of the total film thickness.

### 2.3. Characterization Techniques

A scanning electron microscope (SEM) was used to examine the surface morphology of the titanium implants before being coated with SiC, after being coated with SiC, and after being torqued into and out of an acrylic block. The images were taken using a field-emission SEM (FEI Nova 430, Hillsboro, OR, USA) operating at 5 kV. Within the same tool, energy dispersive spectroscopy (EDS) measurements were taken of the SiC-coated titanium implants using a beam voltage of 10 kV.

A chewing simulator (CS-4.4, SD Mechatronik, Munich, Germany) was used to expose the SiC/Ti interface for SEM examination. The SiC titanium disk was contacted by a steatite ball under a 49 Newton load for 500 cycles at a lateral speed of 30 mm/s and a horizontal movement of 0.7 mm.

Fourier transform infrared spectroscopy (Thermo Electron Magna 760, Waltham, MA, USA) was performed to measure the bulk chemical composition of the SiC on titanium. A specular reflectance apparatus was utilized to measure the SiC composition. 

For the XPS measurements, a Physical Instruments ULVAC PHI system (ULVAC PHI, Kanagawa, Japan) was utilized. The XPS system performed scans using a monochromatic Al 300W X-ray source. The analysis area was 100 µm in diameter. The take-off angle was 50° and the acceptance angle was 7°. The electron pass energy was 23.5 eV for high-resolution scans and 93.5 eV for survey scans. The energy resolution of the system was 0.1 eV and binding energy accuracy was within 0.03 eV. The C 1s core level of adventitious carbon (284.8 eV) was used to calibrate the binding energy for all of the samples examined. 

The surface roughness and topography of SiC-coated and uncoated samples were studied using an atomic force microscopy (AFM) system (Bruker Nanoscope V, Billerica, MA, USA). A silicon AFM probe was operated in tapping mode at a resonance frequency within 200–400 kHz. 

## 3. Results and Discussion

**Figure 2:**
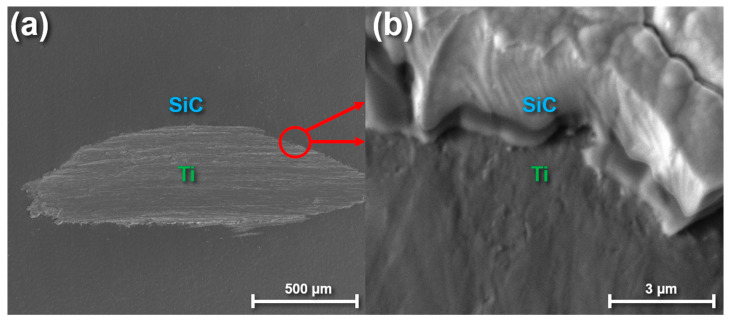
Low-resolution (**a**) and high-resolution (**b**) SEM images of a SiC-coated titanium disk after exposing the SiC/Ti interface.

Prior to coating titanium implants with SiC, high-purity titanium disks were coated with SiC to ensure the coating could conformally cover and adhere to titanium. Figure 2 shows SEM images of a SiC-coated titanium disk after the interface was exposed using a chewing simulator. Figure 2a shows a low-magnification view of the area exposed to the chewing simulator whereas Figure 2b shows a high-magnification image of the SiC/Ti interface on the edge of the exposed region. Dental simulators are traditionally utilized to test the ability of various dental restorative materials to withstand stress [70]. However, since titanium implants do not experience the same chewing stresses as fixed dental prostheses, the main purpose for using a chewing simulator in this study was to expose an interface between the SiC/Ti so that adhesion could be qualitatively examined. As shown in Figure 1, the interface between the titanium surface and SiC coating was continuous, free from noticeable defects, and showed no signs of delamination. The interfacial adhesion was intact and uninterrupted across the entire exposed region. In a previous report, SiC used as a protective coating for dental ceramics was shown to exhibit delamination when placed in a corrosive solution over time, which was mitigated by annealing and plasma treating the SiC after deposition [71]. Similar corrosion/delamination studies should be explored in the future for SiC-coated titanium to further optimize this coating’s protective capability.

**Figure 3:**
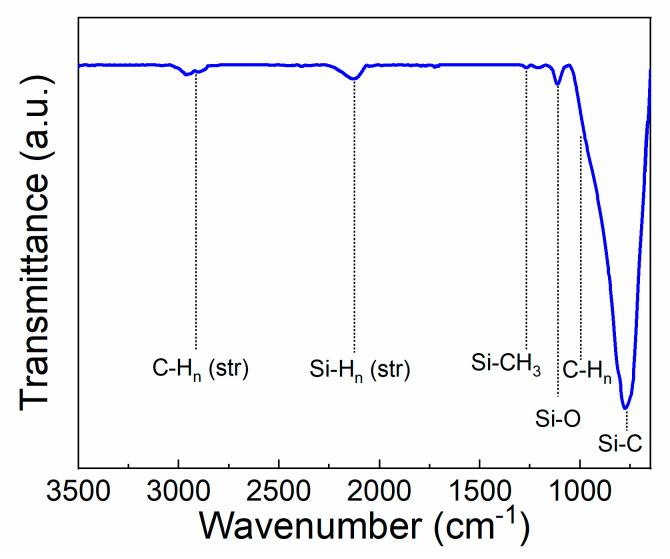
FTIR spectrum taken of SiC on Ti deposited by plasma-enhanced chemical vapor deposition.

After examining the interfacial behavior of the SiC coating on titanium, the SiC film chemistry and composition was explored. Figure 3 illustrates an FTIR spectrum of the deposited SiC on titanium substrates. Titanium substrates coated with 20 nm of SiO_2_ were utilized for background subtraction and baselining the spectrum. A SiO_2_-coated titanium substrate was used for background subtraction so that the bulk chemistry of the SiC coating could be determined with minimal influence of the SiO_2_ layer under it used for adhesion. Despite the use of a SiO_2_-coated titanium reference piece, the measured spectrum may contain some influence from the SiO_2_ adhesion layer which should be considered. The peaks exhibited agree with previous literature reports on PECV-deposited SiC [72,73,74,75,76,77,78]. At 790 cm^−1^, Si-C stretching vibrations correspond to a strong peak [74,75,76]. At 1010 cm^−1^, the shouldered peak corresponds to C-H_n_ wagging. Si-O-Si stretching vibrations are observed at ~1110 cm^−1^. The small peak at 1250 cm^−1^ is due to Si-CH_3_ bond bending [76]. Lastly, the peaks near 2000–2200 cm^−1^ correspond to Si-H_n_ stretching mode vibrations, whereas the peaks at 2890 cm^−1^ are due to C-H_2_ and C=H_3_ stretching mode vibrations [72,73,78]. As shown in the FTIR spectra, some hydrogen related bonds are present within the SiC film. These bonds are commonly found in PECV-deposited SiC and contribute to a lower hardness compared to other methods of SiC deposition [79]. Post-deposition processing such as annealing treatments can be used to reduce the hydrogen incorporation if desired [71]. 

**Figure 4:**
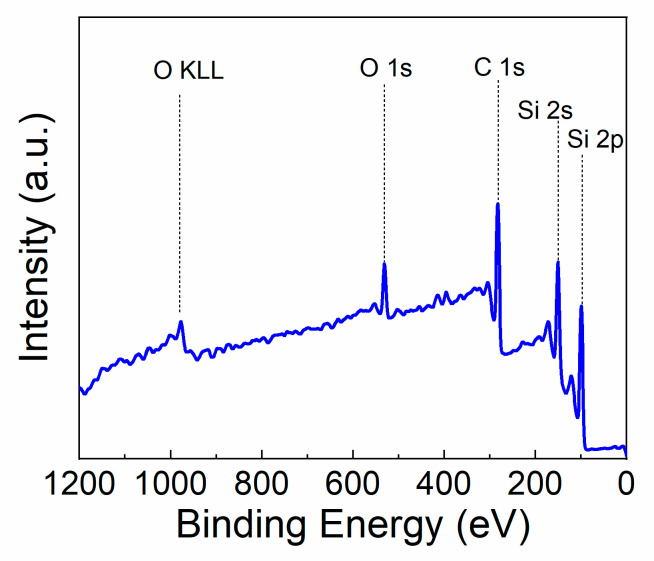
XPS survey scans of SiC on Ti deposited by plasma-enhanced chemical vapor deposition.

After using FTIR to determine the bulk properties of the SiC, XPS survey scans were taken on the SiC-coated titanium disks to quantify the surface composition of the SiC. Figure 4 shows a survey scan of the SiC after coating a titanium substrate. The atomic concentration of the as-deposited SiC surface was measured to include 51% carbon, 38% silicon, 9% oxygen, and 2% nitrogen. The SiO_2_ used as an adhesion layer between the SiC coating and titanium substrate does not affect the XPS data as the penetration depth of XPS analysis is within the first few nanometers of the surface. To measure the bulk composition of the SiC coating, XPS depth profiling was utilized. Table 1 shows the difference in atomic concentration from before ion etching and after ion etching. After two minutes of ion etching using an argon ion beam operating a 2 kV, the oxygen atomic concentration drops from approximately 9% to around 3%, indicating that a surface oxide was present. The nitrogen composition essentially disappears after ion etching whereas the silicon concentration increases to the expected value of PECV-deposited SiC [78,80,81]. Future work should examine how the surface chemistry changes over time, including oxidative and corrosive influences. 

**Figure 5:**
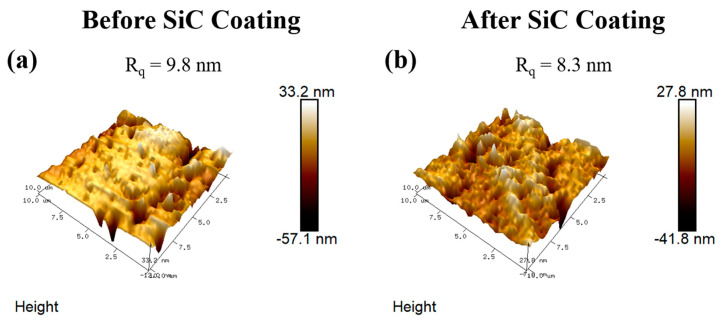
Atomic force microscopy scans comparing the surface roughness of (**a**) uncoated vs. (**b**) SiC-coated substrates.

In order to determine how the SiC coating influenced final surface roughness, AFM measurements were taken on disks before and after SiC deposition and are shown in Figure 5. These disks were initially polished using 600 grit sandpaper to mimic the surface roughness of the titanium implants. Six samples were averaged for each condition using an analysis area of 50 µm × 50 µm for each sample. Prior to SiC coating, the root mean square average of height deviation (R_Q_) was determined to be 9.8 ± 1.93 nm. After these same substrates were coated with SiC, the measured R_Q_ was 8.3 ± 1.72 nm. The minor change in surface roughness after SiC deposition indicates that some planarization does occur due to the coating process but also shows that the general substrate topography is maintained. In the future, additional work should focus on quantifying changes in surface roughness as a function of coating thickness, deposition technique, and deposition parameters. 

**Figure 6:**
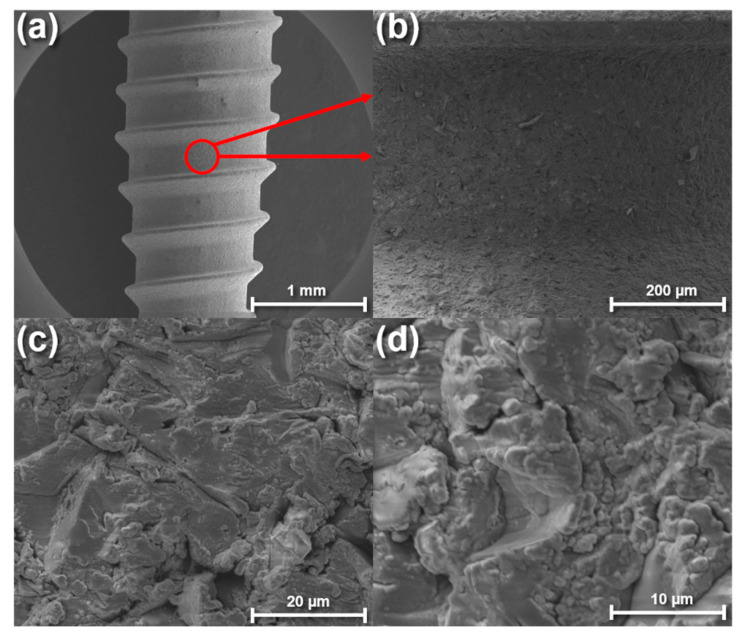
Scanning electron microscope images of a SiC-coated titanium implant at various magnifications. Image (**a**) shows the overall surface of the implant, whereas images (**b**–**d**) show detailed images of the implant surface morphology at increasing magnifications.

After characterizing the chemical properties of the deposited SiC, we then proceeded to coat titanium implants with SiC to study the coating’s conformality and adhesive properties. Figure 6 shows SEM images of the titanium implant after being coated. Since most of the commercially available titanium implants are purposely roughened to enhance osteointegration by acid-etching and/or grit-blasting [17,18,40,41], we wanted to ensure the deposited SiC (200 nm) could conformably coat the surface while minimally affecting the surface topography. As shown in Figure 6, the SiC coating was able to conform to the titanium implant and maintain the original surface topography set by the implant manufacturer. The maintained surface topography shown in these images agrees with the previously mentioned AFM results.

**Figure 7:**
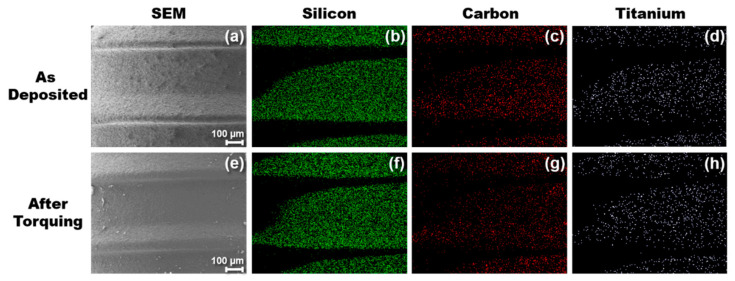
Surface composition of a SiC-coated titanium implant measured using energy dispersive spectroscopy. Images (**a**–**d**) were taken of the SiC-coated implant directly after fabrication, whereas images (**e**–**h**) were taken after torquing into an acrylic block used to simulate clinical implant placement.

After torquing the SiC-coated implant into the PMMA blocks and then removing the implant, SEM and EDS images were taken to determine if the SiC coating remained intact. Figure 6 shows before and after elemental mapping images of the SiC-coated implant taken by EDS. At a beam voltage of 10 kV, the penetration depth of the X-rays was between 0.75–1 µm. Elemental scans were taken at various thread locations on the implant and confirmed that the coating adhered to the titanium during the torquing process. All elements (silicon shown in green, carbon shown in red, and titanium shown in white) exhibited no major change in concentration or spatial arrangement after the implant was placed into the PMMA block. The dark curved areas in Figure 7 that do not appear colored are due to the implant thread shape inhibiting signal collection by the EDS detector. 

Table 2 details the average atomic percentages of various elements before and after torquing the implant into the PMMA. Elemental scans were taken across the implant to ensure the adhesion results were uniform across the surface. The atomic concentration of carbon increased marginally from 46% to 49%. This increase could be due to residual PMMA particles on the SiC surface after removing the implant from the blocks. The silicon concentration decreased slightly from 36% to 33%. The decrease is likely due to a higher amount of carbon on the surface along with slight surface oxidation due to the creation of silicon dangling bonds during the torquing process. The atomic concentration of oxygen increased from 7% to 9% after torquing. The atomic concentration of titanium remained constant at approximately 5%, whereas the aluminum concentration decreased minimally from 5% to 4%. Titanium and aluminum were monitored to determine if any area of the SiC delaminated during the implant placement process since titanium makes up the bulk of the implant, whereas aluminum is present due to the grit-roughening process performed by the manufacturer. Unlike XPS, the depth resolution of EDS can probe microns deep into a sample, explaining the presence of Ti and Al under the SiC coating. From the EDS measurements and mapping, it is evident that the SiC coating remained intact across the surface of the titanium implant after torquing. It is worth noting that the accuracy of the EDS tool utilized can be up to 5% for major elements and between 1–2% when measuring the same sample in different sessions. Therefore, the main takeaway from the EDS results is qualitative in nature and shows that the SiC coating maintained its adherence to Ti and remained chemically stable after simulated implant placement.

## 4. Conclusions

We have shown that PECV-deposited SiC for the use of a protective coating can conformably coat titanium implants and remain adhered to the surface after implant placement into PMMA. A chewing simulator was used to expose the interface of a SiC-coated titanium disk and SEM revealed that the interface between the titanium surface and SiC coating did not show any noticeable defects or delamination. The use of XPS and FTIR confirmed SiC uniformity between the bulk and surface of the coating. SEM images at various magnifications illustrated that the microscale topography of the implant surface remained roughened and uniform after PECVD SiC deposition. From the EDS measurements and mapping, it is evident that the SiC coating remained intact across the surface of the titanium implant after torquing. Despite the promising results of this study, there is still significant work needed before SiC can be proven as a viable coating for implant technology. Although previous work has shown favorable biocompatibility for SiC [57,58,59,60,61,62,63], application-specific in vivo studies must be completed. Additionally, peel-off tests and other adhesive measures should be performed to quantify and optimize the SiC adhesion onto titanium. 

## Figures and Tables

**Table 1:** Atomic concentration determined by X-ray photoelectron spectroscopy for the SiC-coated titanium disks before and after ion etching.

Element	No Ion Etching (At %)	After Ion Etching for 2 min (At %)
Carbon	51	49
Silicon	38	48
Oxygen	9	3
Nitrogen	2	<1

**Table 2:** Atomic concentration determined by energy dispersive spectroscopy for the SiC-coated titanium implants before and after torquing.

Element	As Deposited (At %)	After Torquing (At %)
Carbon	46	49
Silicon	37	33
Oxygen	7	9
Titanium	5	5
Aluminum	5	4
